# Prognostic Performance of Existing Scoring Systems among Critically Ill Patients Requiring Continuous Renal Replacement Therapy: An Observational Study

**DOI:** 10.3390/jcm10194592

**Published:** 2021-10-06

**Authors:** Chieh-Li Yen, Pei-Chun Fan, George Kuo, Cheng-Chia Lee, Jia-Jin Chen, Tao-Han Lee, Yi-Ran Tu, Hsiang-Hao Hsu, Ya-Chung Tian, Chih-Hsiang Chang

**Affiliations:** 1 Kidney Research Center, Department of Nephrology, Chang Gung Memorial Hospital, Taoyuan 33305, Taiwan; b9102087@yahoo.com.tw (C.-L.Y.); franwis1023@gmail.com (P.-C.F.); b92401107@gmail.com (G.K.); a12490@cgmh.org.tw (C.-C.L.); raymond110234@hotmail.com (J.-J.C.); kate0327@hotmail.com (T.-H.L.); yirantu1020@gmail.com (Y.-R.T.); hsianghao@gmail.com (H.-H.H.); dryctian@yahoo.com (Y.-C.T.); 2 College of Medicine, Chang Gung University, Taoyuan 33305, Taiwan

**Keywords:** ICU, CRRT, CVVH, SOFA, APACHE, old age

## Abstract

Background: Among critical patients, few studies have evaluated the discrimination of current illness scoring systems in predicting outcomes after continuous renal replacement therapy (CRRT) initiation. Methods: Patients receiving CRRT in the ICU between 2005 and 2018 from the Chang Gung Research Database were extracted. All the components of the Acute Physiology Assessment and Chronic Health Evaluation (APACHE) III, Sequential Organ Failure Assessment (SOFA), qSOFA, and MOSAIC scoring systems on days 1, 3, and 7 of CRRT were recorded. Patients older than 80 years were identified and analyzed separately. Results: We identified 3370 adult patients for analysis. The discrimination ability of the scoring systems was acceptable at day 7 after CRRT initiation, including SOFA (area under the receiver operating characteristic curve, 74.1% (95% confidence interval, 71.7–76.5%)), APACHEIII (74.7% (72.3–77.1%)), and MOSAIC (71.3% (68.8%–73.9%)). These systems were not ideal on days 1 and 3, and that of qSOFA was poor at any time point. The discrimination performance was slightly better among patients ≥80 years. Conclusions: APACHE III, MOSAIC, and SOFA can be intensivists and families’ reference to make their decision of withdrawing or withholding CRRT after a short period of treatment, especially in adults ≥80 years old.

## 1. Introduction

Acute kidney injury (AKI) is a common but serious complication in critically ill patients requiring treatment in intensive care units (ICUs) [1,2]. AKI is a part of multiple organ failure syndrome and results in several challenging conditions, such as hyperkalemia, fluid accumulation, and severe acidosis; accordingly, the prognosis of patients with AKI has remained poor, despite advances in ICU care [3,4]. Among these AKI populations, administration of continuous renal replacement therapy (CRRT), because of its gentle but continuous fluid clearance and better hemodynamic tolerance, caused renal function to be preserved, particularly in those with unstable hemodynamic status and poor fluid control [5,6]. Along with the increasing age and severity of ICU populations, the incidence of AKI requiring CRRT has been increasing considerably [7,8]. As CRRT-associated resources are not unlimited, the incremental need of CRRT imposes a burden on the ICU system [9]. Moreover, even with appropriate CRRT, the mortality rate of these patients remains very high, ranging between 40% and 80% [10,11]. Thus, for better allocation of CRRT-associated resources and modification of treatment strategies, a reliable model to assess conditions and predict outcomes of severely ill patients with CRRT requiring AKI is urgently required, especially in older people (such as those older than 80 years). Considering the poor prognosis and shorter life expectancy, conservative treatment with less invasive procedures is always a more favorable option for this population [12]. A precise assessment model may therefore help patients and families in decision making regarding choosing conservative treatment or switching to it after CRRT initiation.

Several popular illness-scoring systems exist for identifying high-risk ICU patients independent of their underlying disease processes, including the Acute Physiology Assessment and Chronic Health Evaluation system (APACHE), the Sequential Organ Failure Assessment (SOFA), and quick SOFA (qSOFA) systems. The APACHE system is a comprehensive assessment of different physiologic indexes, but its complex algorithms limit its application in daily practice [13,14]. The SOFA score system was initially introduced to assess the severity of organ failure among patients with sepsis and was then expanded to other critical diseases [15,16,17]. The qSOFA system was introduced to be an easy predictive tool for patients with suspected infection [18]. In addition, Kim et al., in their study involving an 828-patient cohort, developed a new system, the Mortality Scoring system for AKI with CRRT (MOSAIC), to predict mortality in patients undergoing CRRT [19]. Although these predictive systems have been validated in several ICU populations, they have not been effectively tested in people with AKI requiring CRRT or have only been tested in small cohorts.

Therefore, in this study, we used a large comprehensive medical database, the Chang Gung Research Dataset (CGRD), to assess the performance of several scores on predicting mortality in patients with AKI requiring CRRT and in patients older than 80 years with AKI requiring CRRT.

## 2. Material and Methods

### 2.1. Data Source

The CGRD is based on medical information from Taiwan’s largest hospital network, Chang Gung Memorial Hospital system. It includes the detailed laboratory and physical information of more than 5000 ICU patients receiving CRRT between 2005 and 2018. The Chang Gung Memorial Hospital system comprises four tertiary medical centers and three teaching hospitals and covers approximately 10% of the annual medical services of Taiwan, including approximately 20,000 ICU patients per year [20,21]. The CGRD is a deidentified dataset based on the electronic medical records of the Chang Gung Memorial Hospital system. It contains comprehensive medical records, including ICU admission, inpatient orders, medication prescriptions, procedure interventions, laboratory data, and examination reports, thus making the database suitable for assessing the application of illness predictive systems. Moreover, any information that can identify a specific person in the CGRD is scrambled before releasing the data for research purposes, and de-identified consistent data encryption is used to link medical information. Thus, this study was approved with a waiver of the need for consent from the Institutional Review Board of Chang Gung Medical Foundation (approval number: 201900835B0).

### 2.2. Study Population

**Figure 1:**
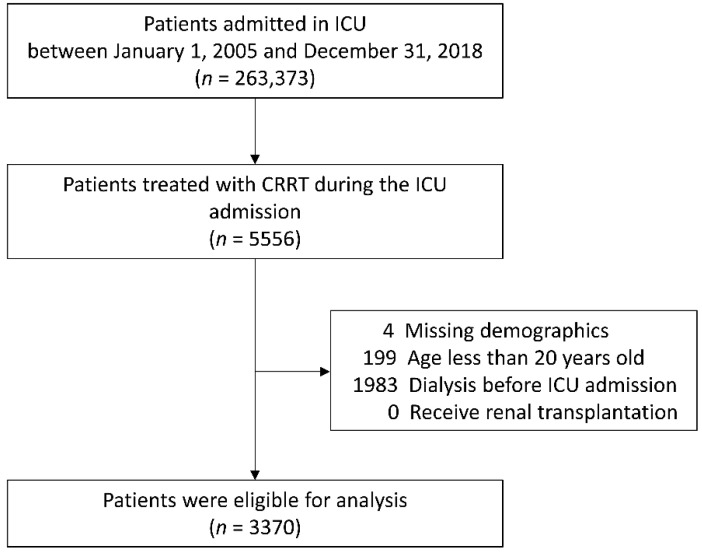
Flowchart of patient selection. ICU, intensive care unit; CRRT, continuous renal replacement therapy.

All ICU admissions with CRRT between 2005 and 2018 in CGRD were identified. The first episode of admission was considered an index admission if a patient experienced two or more ICU admissions during the study period. Patients who received RRT or dialysis prior to the index admission were excluded. Patients who were younger than 20 years old or had missing demographic data (age and sex) were also excluded. In addition, a subgroup of patients older than 80 years was identified to better understand the application of the illness predictive systems in this age group (Figure 1).

### 2.3. Clinical Parameters and Outcomes

All the components of APACHE III, SOFA, qSOFA, and MOSAIC systems on days 1, 3, and 7 of CRRT initiation were retrospectively collected from the CGRD. While these scores were generated according to the collected information, the missing variables at day 1 of CRRT were imputed as normal as the default [22,23]. Consequently, the missing variables at days 3 and 7 of CRRT were imputed as the last available data or as normal if no data were available. The baseline comorbidities were detected based on two or more outpatient diagnoses prior to the index admission. Demographic data, laboratory data, and use of mechanical ventilators or inotropic agents were recorded according to medical records on the day of CRRT initiation.

The outcomes of interest were 3-day and 7-day mortality after CRRT initiation, ICU mortality, and in-hospital mortality. The length of CRRT, ICU stay, and hospitalization were also measured from the day of CRRT initiation until the end of CRRT, transfer, discharge, or mortality.

### 2.4. Statistical Analysis

The characteristics of the patients who died and who survived during the index hospitalization were compared using a chi-square test for categorical variables or an independent-samples *t* test for continuous variables. The ability of the existing systems to predict mortality was assessed using the area under the receiver operating characteristic curve (AUC), with standard error calculated using DeLong’s nonparametric method. Performance was considered acceptable when the AUC was ≥70% [24]. The optimal cutoffs were further determined using the Youden index when AUC was >70%. A two-sided *p* value of <0.05 was considered statistically significant. Statistical analyses were performed using MedCalc 19 (MedCalc Software, Ostend, Belgium).

## 3. Results

### 3.1. Patient Characteristics

We identified 3370 patients with new-onset AKI requiring CRRT during ICU admission between 2005 and 2018 from the CGRD. As presented in Table 1, the mean age of the patients was 64.1 years (standard deviation [SD] = 15.7 years), and 67.7% (*n* = 2283) were men. Regarding baseline comorbidities, the mean Charlson comorbidity index was 4.4 (SD = 3.3), and 67.3% (*n* = 2268) had chronic kidney disease. Of them, 28.5% (*n* = 960) of patients were admitted to the surgical ICU, whereas the others were admitted to the medical ICU. The mean serum creatinine and blood urea nitrogen on the day of CRRT initiation were 4.0 mg/dL (SD = 2.2 mg/dL) and 68.6 mg/dL (SD = 43.3 mg/dL), respectively. Most patients were under treatment with mechanical ventilators (94.9%, *n* = 3199) or inotropic agents (96.1%, *n* = 3240) at CRRT initiation. The median duration of CRRT, ICU stay, and hospitalization was 3 days (interquartile range (IQR): 2–6 days), 9 days (IQR: 4–19 days), and 16 days (IQR: 6–35 days), respectively. Furthermore, 37.1% of patients died within 3 days after CRRT initiation, 50.2% died within 7 days, 67.5% died in the ICU, and 71.9% of patients died during hospitalization (Table 1).

### 3.2. Prediction of 3-Day and 7-Day Mortality after CRRT

All four systems exhibited poor performance in predicting 3-day mortality on the first day of CRRT initiation, with AUCs near or below 60% (Supplemental Table S1). The results were similar when the cohort was restricted to patients older than 80 years. Table 2 presents the results of predicting 7-day mortality after CRRT by the scoring systems at days 1 and 3 of CRRT initiation. The systems exhibited poor performance in predicting 3-day mortality on day 1, but the performance improved on day 3, especially for APACHE III in both the whole cohort (AUC 76.1%, 95% confidence interval (CI), 73.6–78.5%) and in patients older than 80 years (AUC 78.9%; 95% CI, 73.1–84.8%). The optimal cutoffs and the corresponding sensitivity and specificity are provided in Supplemental Table S2.

### 3.3. Prediction of ICU Mortality and In-Hospital Mortality

Table 3 displays the results of predicting ICU mortality on days 1, 3, and 7 of CRRT initiation. No scoring systems had satisfactory performance on days 1 and 3 after CRRT initiation, with all the AUCs being less than 70%. However, the performance of predicting ICU mortality improved on day 7 for SOFA (AUC, 74.1%, 95% CI, 71.7–76.5%), APACHE III (AUC, 74.7%, 95% CI, 72.3–77.1%), and MOSAIC (AUC, 71.3%, 95% CI, 68.8–73.9%), whereas that of qSOFA remained unsatisfactory, with AUCs <70% in the whole population or in patients older than 80 years (Table 3).

Table 4 presents the results of predicting in-hospital mortality on days 1, 3, and 7 of CRRT initiation. Similar to the results of ICU mortality, the performance was acceptable on day 7 for SOFA (AUC, 71.8%, 95% CI, 69.3–74.3%) and APACHE III (AUC, 73.5%, 95% CI, 71.0–76.0%). For MOSAIC, the performance on day 7 was poor for the whole cohort but was satisfactory when only patients older than 80 years were considered (AUC, 74.7%, 95% CI, 67.9–81.4%) (Table 4).

**Figure 2:**
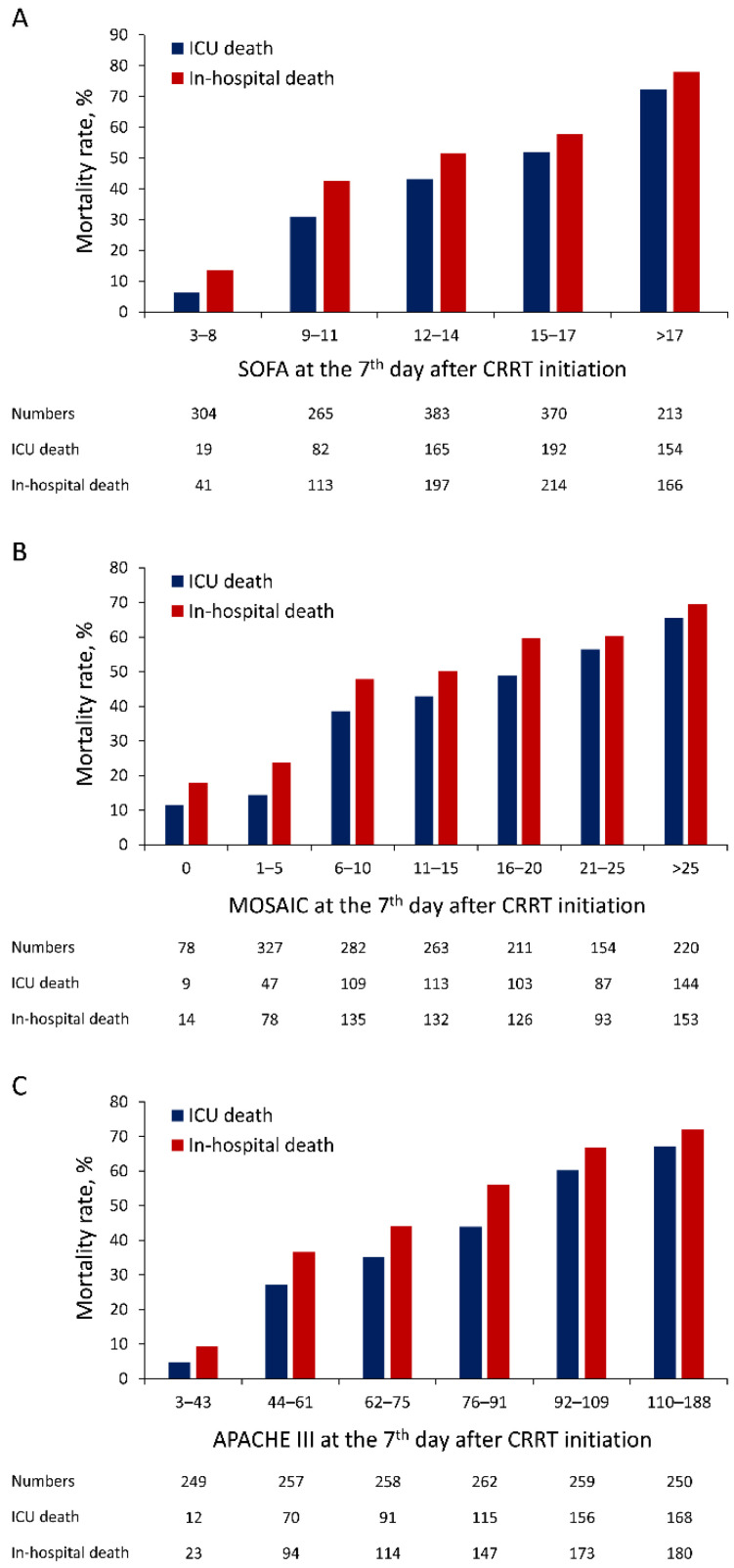
Distribution of SOFA (**A**), MOSAIC (**B**), and APACHE III (**C**) scores at day 7 after CRRT initiation and their relationship with ICU mortality and in-hospital mortality in the whole population. SOFA, Sequential Organ Failure Assessment; APACHE, Acute Physiology and Chronic Health Evaluation; MOSAIC, Mortality Scoring system for AKI with CRRT.

**Figure 3:**
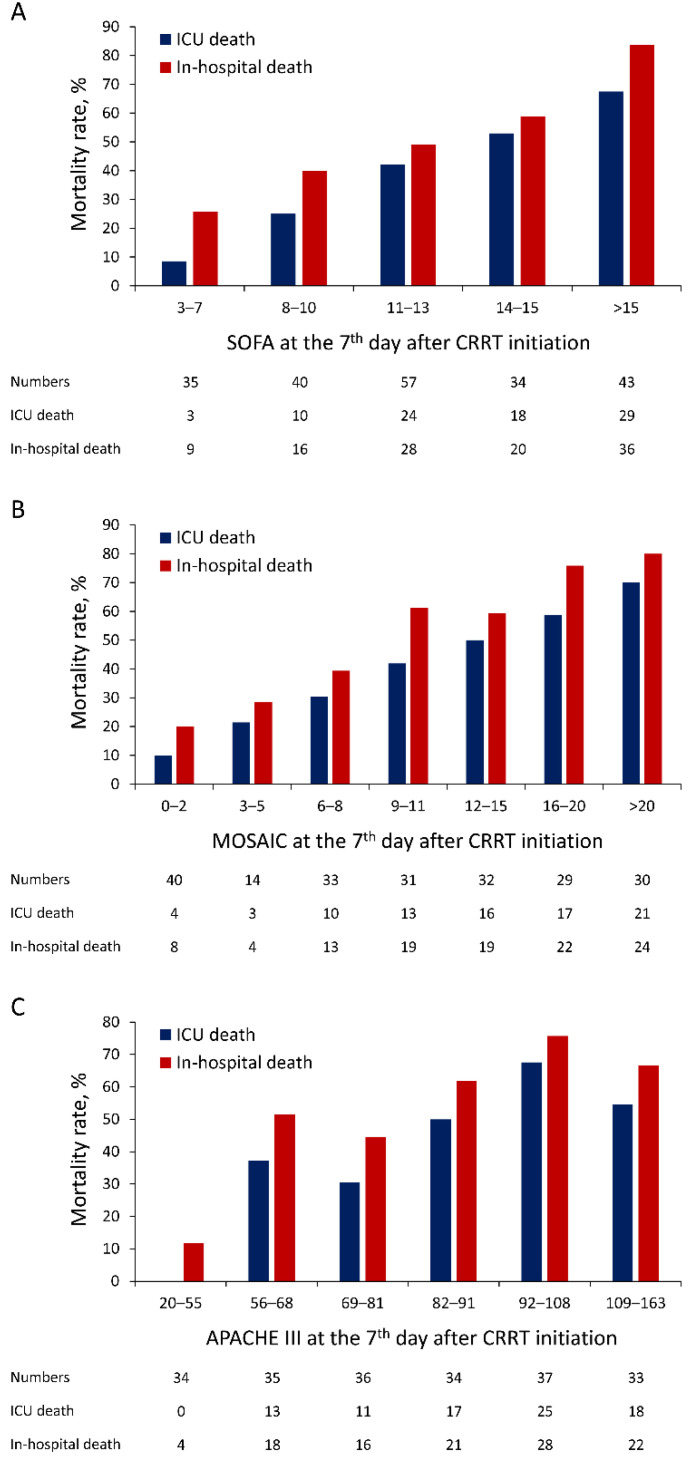
Distribution of SOFA (**A**), MOSAIC (**B**), and APACHE III (**C**) scores at day 7 after CRRT initiation and their relationship with ICU mortality and in-hospital mortality in patients older than 80 years.

The optimal cutoffs and the corresponding sensitivity and specificity are provided in Supplemental Table S2. The distributions of SOFA, MOSAIC, and APACHE III scores on day 7 after CRRT initiation and their relationship with ICU mortality and in-hospital mortality in the whole cohort (Figure 2A–C) and in patients older than 80 years are illustrated (Figure 3A–C).

## 4. Discussion

CRRT has become a crucial treatment in ICU. In this study, around 2.1% of total ICU patients had received CRRT before, which was similar to previous research studies in France (4.3%) [25] and Korea (3.3%) [8]. Although illness scoring systems have been used for assessing severity and predicting mortality among ICU populations [16,23], few studies have evaluated their use in allocating limited CRRT resources and helping families opting for or switching to conservative treatment before or during CRRT if poor prognosis is predicted, which is especially crucial in older people. The current large observational study evaluated the prognostic performance of four current scoring systems in predicting outcomes after CRRT initiation and demonstrated that SOFA, APACHE III, and MOSAIC can satisfactorily predict ICU or in-hospital mortality only on day 7, with AUCs for ICU mortality being 74.1, 74.1, and 71.3, respectively, and optimal cutoffs being 14, 82, and 10, respectively. For short-term outcomes (7-day mortality), only APACHE III on day 3 was found to have a good discrimination (AUC = 76.1), with the optimal cutoff being 107. Unlike previous studies assessing the predictive accuracy of scoring systems on the first day of ICU admission [26] or diagnosis of sepsis [27], our data indicated that the use of these systems on the first day of CRRT initiation was unsatisfactory for predicting short-term or long-term outcomes. Thus, we speculate that the key factor influencing the survival of these critically ill patients with AKI depends on whether their physiological function improves after CRRT initiation, such as hyperkalemia-induced arrhythmia and fluid overload-induced respiratory failure, or whether renal failure is a part of progressive multiple organ dysfunction. It is challenging to determine these two situations by using only scoring systems. In other words, SOFA, APACHE III, and MOSAIC are more suitable for assessing outcomes after a few days of CRRT initiation rather than for deciding whether to initiate CRRT. Among the three models, APACHE III was noted to be the best at predicting short-term; however, its complex algorithms and non-superior prediction of ICU mortality or in-hospital mortality make it less attractive in daily ICU practice. MOSAIC, which is a combination of part of SOFA and APACHE II [19], was proven to have a noninferior prognostic performance compared with APACHE III or SOFA. However, the lack of other proven utilities limits its use in critical care. Simplicity and ease of use are the most crucial advantages of the SOFA score [28,29], and its discrimination is equivalent to other complex models. In addition to the CRRT population, SOFA’s good prognostic performance has been proven in several scenarios of ICU care, such as sepsis [27], or cardiac intensive care units [23], which makes it more attractive for use in daily care. The prognostic performance of qSOFA in predicting mortality amount CRRT patients, however, was found to be unsatisfactory in all analyses in the present study, similar to previous research [16]; this indicates that qSOFA might not be appropriate for predicting outcomes in critically ill patients.

In developed countries, patients older than 80 years may be the fastest expanding subgroup in ICU populations [30]. However, these patients have many comorbidities, exhibit a decline in organ function, and are frail, leading to extremely high in-hospital mortality and after-discharge mortality among these populations compared with their younger counterparts [31,32]. Although old age is an independent risk factor for AKI development among the ICU population, no large-scale study has evaluated outcome prediction in patients older than 80 years requiring CRRT. In the present study, we found that SOFA, APACHE III, and MOSAIC had better prediction performance in patients older than 80 years than in the whole cohort, with AUCs for ICU mortality being 75.4, 72.6, and 74.2, respectively, and optimal cutoffs being 13, 85, and 13, respectively. The day 3 APACHE III and SOFA models had acceptable prediction performance for ICU mortality and in-hospital mortality. Notably, in patients older than 80 years, the day 7 SOFA and MOSAIC had better prediction performance of ICU mortality and in-hospital mortality than APACHE III. This might be attributed to the lower rates of residual vital organ function in this patient subgroup and the lower probability of organ function recovery after injury. Thus, among patients older than 80 years, the index of organ failure, such as SOFA and MOSAIC, may surpass the physical index APACHE III in predicting ICU or in-hospital mortality. Further research is warranted to validate these findings. Thus, these scoring systems may help patients older than 80 years, their families, and intensivists make decisions to withdraw CRRT if the prognosis is determined to be poor, which may save medical costs, and notably, reduce suffering.

This study had the strength of a large sample size from multiple centers. Moreover, this was the first study to evaluate the prediction of outcomes among critically ill patients older than 80 years requiring CRRT. Nevertheless, several limitations of this study should be acknowledged. First, detailed information on CRRT prescriptions, such as dialysate dose, blood flow rate, and anticoagulation, was not available in the CGRD. Second, the observational design precluded the determination of causal relationships or analysis of long-term outcomes after discharge. Third, the missing variables were imputed as normal as the default or imputed as the last available data since CRRT initiation in this study, which may lead to an underestimation of mortality risk. In addition, imputing missing variables as the last available data may lead to discrepancies in the time the data were obtained, which may further reduce the accuracy of illness score systems. Fourth, in this study, real serum creatinine was used to calculate illness scores instead of setting these renal components of scoring systems to maximum after initiation of CRRT, which could simplify the calculations for intensivists but would inevitably underestimate the entire score values.

## 5. Conclusions

We comprehensively reviewed the application of four popular illness scoring systems in predicting outcomes of ICU patients with AKI requiring CRRT by using the largest cohort thus far. We demonstrated that APACHE III, MOSAIC, and SOFA scores 7 days after CRRT initiation favorably discriminated ICU mortality and in-hospital mortality. In patients older than 80 years, the prognostic performances of these scoring systems were even better. Although these scoring systems cannot be used to decide whether to initiate CRRT or not, they can be provided for intensivists and families’ reference to make their decisions on withdrawing or withholding the CRRT after a short period of treatment, which could help shorten the suffering of patients with poor prognosis and allocate more resources to patients to whom the CRRT may be more beneficial.

## Figures and Tables

**Table 1:** Patient characteristics according to the survival status at the day of discharge from the index hospitalization.

Variable	Valid Number	Total (*n* = 3370)	Survivor (*n* = 946)	Non-Survivor (*n* = 2424)	*p* Value
Demographics					
Age, year	3370	64.1 ± 15.7	61.8 ± 15.7	65.0 ± 15.6	<0.001
Male	3370	2283 (67.7)	652 (68.9)	1631 (67.3)	0.361
Body mass index, kg/m^2^	2883	27.1 ± 25.0	27.7 ± 22.0	26.9 ± 26.0	0.412
Comorbidity					
Heart failure	3370	834 (24.7)	217 (22.9)	617 (25.5)	0.128
Coronary atrial disease	3370	861 (25.5)	210 (22.2)	651 (26.9)	0.005
Chronic obstruction pulmonary disease	3370	537 (15.9)	137 (14.5)	400 (16.5)	0.150
Asthma	3370	273 (8.1)	70 (7.4)	203 (8.4)	0.351
Liver cirrhosis	3370	638 (18.9)	128 (13.5)	510 (21.0)	<0.001
Stroke	3370	350 (10.4)	94 (9.9)	256 (10.6)	0.593
Diabetes mellitus	3370	1177 (34.9)	344 (36.4)	833 (34.4)	0.274
Hypertension	3370	1610 (47.8)	429 (45.3)	1181 (48.7)	0.078
Chronic kidney disease	3370	2268 (67.3)	579 (61.2)	1689 (69.7)	<0.001
Malignancy	3370	1179 (35.0)	292 (30.9)	887 (36.6)	0.002
Charlson’s Comorbidity Index score	3370	4.4 ± 3.3	3.8 ± 3.3	4.6 ± 3.3	<0.001
Route of ICU	3370				0.003
Surgical		960 (28.5)	304 (32.1)	656 (27.1)	
Medical		2410 (71.5)	642 (67.9)	1768 (72.9)	
Laboratory data at initiation of CRRT					
Creatinine, mg/dL	3332	4.0 ± 2.2	4.3 ± 2.4	3.9 ± 2.2	<0.001
Blood urea nitrogen, mg/dL	3296	68.6 ± 43.3	66.7 ± 42.1	69.3 ± 43.7	0.123
Hemoglobin, g/dL	3279	9.9 ± 2.5	10.1 ± 2.4	9.9 ± 2.5	0.090
Platelet, count ×10^3^	3216	111.3 ± 93.4	118.7 ± 96.6	108.5 ± 92.0	0.005
Albumin, mg/dL	2352	2.4 ± 0.6	2.5 ± 0.6	2.4 ± 0.6	<0.001
pH	2900	7.27 ± 0.17	7.28 ± 0.17	7.26 ± 0.17	0.014
Treatment at initiation of CRRT					
Mechanical ventilator	3370	3199 (94.9)	890 (94.1)	2309 (95.3)	0.162
Inotropic agent	3370	3240 (96.1)	874 (92.4)	2366 (97.6)	<0.001
Days from ICU admission to CRRT	3370	3 (2,5)	2 (2, 5)	3 (2, 6)	0.023
In-hospital outcome					
Duration of CRRT, day	3370	3 (2,6)	-	-	-
Duration of ICU stay, day	3370	9 (4,19)	-	-	-
Duration of hospitalization, day	3370	16 (6,35)	-	-	-
Death within 3 days after CRRT	3370	1251 (37.1)	-	-	-
Death within 7 days after CRRT	3370	1693 (50.2)	-	-	-
Death during ICU admission	3370	2276 (67.5)	-	-	-

ICU, intensive care unit; CRRT, continuous renal replacement therapy.

**Table 2:** Performance of the scoring systems in predicting 7-day mortality since CRRT initiation in the entire cohort and in patients older than 80 years.

Day/Score	Total Cohort			Cohort with Octogenarian (Age ≥ 80 Years)		
	Survivor (*n* = 1677)	Non-Survivor (*n* = 1693)	AUC, % (95% CI)	Survivor (*n* = 231)	Non-Survivor (*n* = 327)	AUC, % (95% CI)
Day 1 ^a^ (*n* = 3370)						
SOFA	14.1 ± 3.4	14.9 ± 3.3	56.5 (54.6–58.4)	13.0 ± 3.2	13.8 ± 3.1	55.7 (50.9–60.5)
qSOFA	1.9 ± 0.8	2.1 ± 0.8	56.0 (54.2–57.8)	1.9 ± 0.8	2.1 ± 0.7	55.0 (50.4–59.6)
APACHE III	97.3 ± 28.7	110.8 ± 28.2	63.2 (61.3–65.1)	100.9 ± 27.3	111.6 ± 27.1	61.6 (56.9–66.3)
MOSAIC	19.2 ± 10.4	23.6 ± 11.1	61.4 (59.5–63.3)	16.7 ± 9.9	20.1 ± 10.8	58.8 (54.0–63.5)
Day 3 ^b^ (*n* = 2119)						
SOFA	14.0 ± 3.5	15.7 ± 3.0	63.9 (61.1–66.7)	12.7 ± 3.5	14.3 ± 3.2	63.5 (56.9–70.1)
qSOFA	1.6 ± 0.8	2.2 ± 0.7	68.7 (66.2–71.3)	1.6 ± 0.7	2.3 ± 0.7	74.3 (68.8–79.7)
APACHE III	87.0 ± 28.6	115.4 ± 28.2	76.1 (73.6–78.5)	88.2 ± 24.3	119.7 ± 30.1	78.9 (73.1–84.8)
MOSAIC	17.5 ± 9.9	24.1 ± 10.7	67.7 (64.9–70.6)	14.5 ± 8.4	20.1 ± 11.1	64.4 (57.3–71.5)

CRRT, continuous renal replacement therapy; AUC, area under the curve; CI, confidence interval; SOFA, Sequential Organ Failure Assessment; qSOFA, quick Sequential Organ Failure Assessment; APACHE, Acute Physiology and Chronic Health Evaluation; MOSAIC, Mortality Scoring system for AKI with CRRT. ^a^: the missing components of the score system were imputed by a score of 0; ^b^: the missing components of the score system were imputed using the score at day 1 at CRRT; otherwise, they were imputed by a score of 0.

**Table 3:** Performance of the scoring systems in predicting ICU mortality in the entire cohort and in patients older than 80 years.

Day/Score	Total Cohort			Cohort with Octogenarian (Age ≥ 80 Years)		
	Survivor (*n* = 1094)	Non-Survivor (*n* = 2276)	AUC (95% CI)	Survivor (*n* = 151)	Non-Survivor (*n* = 407)	AUC (95% CI)
Day 1 ^a^ (*n* = 3370)						
SOFA	13.7 ± 3.4	14.8 ± 3.3	59.2 (57.2–61.2)	12.6 ± 3.4	13.8 ± 3.1	59.9 (54.6–65.2)
qSOFA	1.9 ± 0.8	2.0 ± 0.8	55.5 (53.6–57.4)	1.9 ± 0.8	2.0 ± 0.8	53.4 (48.4–58.5)
APACHE III	95.5 ± 29.4	108.2 ± 28.2	62.3 (60.3–64.4)	98.7 ± 27.5	110.3 ± 27.1	62.3 (57.1–67.6)
MOSAIC	18.8 ± 10.7	22.7 ± 11.0	60.1 (58.1–62.2)	16.5 ± 10.1	19.5 ± 10.7	58.0 (52.6–63.3)
Day 3 ^b^ (*n* = 2119)						
SOFA	13.4 ± 3.5	15.4 ± 3.1	66.1 (63.8–68.5)	11.8 ± 3.4	14.2 ± 3.2	69.2 (63.4–75.1)
qSOFA	1.6 ± 0.8	1.9 ± 0.8	61.2 (58.9–63.4)	1.5 ± 0.8	2.0 ± 0.7	66.3 (60.6–71.9)
APACHE III	83.4 ± 29.5	102.5 ± 29.1	67.8 (65.5–70.1)	85.7 ± 24.7	106.8 ± 30.2	69.9 (64.1–75.8)
MOSAIC	16.9 ± 10.3	20.9 ± 10.3	61.7 (59.3–64.1)	13.6 ± 8.5	18.2 ± 10.0	63.0 (56.7–69.2)
Day 7 ^c^ (*n* = 1677)						
SOFA	11.0 ± 4.7	14.9 ± 3.4	74.1 (71.7–76.5)	10.2 ± 4.1	14.0 ± 3.3	75.4 (68.9–81.8)
qSOFA	1.2 ± 0.9	1.8 ± 0.8	66.7 (64.2–69.2)	1.4 ± 0.8	1.8 ± 0.8	61.6 (54.4–68.8)
APACHE III	65.0 ± 31.7	93.1 ± 27.3	74.7 (72.3–77.1)	72.9 ± 28.0	94.6 ± 23.0	72.6 (65.8–79.3)
MOSAIC	10.6 ± 9.2	17.9 ± 10.0	71.3 (68.8–73.9)	8.7 ± 7.2	15.5 ± 8.5	74.2 (67.5–80.9)

CRRT, continuous renal replacement therapy; AUC, area under the curve; CI, confidence interval; SOFA, Sequential Organ Failure Assessment; qSOFA, quick Sequential Organ Failure Assessment; APACHE, Acute Physiology and Chronic Health Evaluation; MOSAIC, Mortality Scoring system for AKI with CRRT. ^a^: the missing components of the score system were imputed by a score of 0; ^b^: the missing components of the score system were imputed using the score at day 1 at CRRT; otherwise, they were imputed by a score of 0; ^c^: the missing components of the score system were imputed using the score at day 3 at CRRT; otherwise, they were imputed by a score of 0.

**Table 4:** Performance of the scoring systems in predicting in-hospital mortality in the entire cohort and in patients older than 80 years.

Day/Score	Total Cohort			Cohort with Octogenarian (Age ≥ 80 Years)		
	Survivor (*n* = 946)	Non-Survivor (*n* = 2424)	AUC (95% CI)	Survivor (*n* = 122)	Non-Survivor (*n* = 436)	AUC (95% CI)
Day 1 ^a^ (*n* = 3370)						
SOFA	13.7 ± 3.4	14.8 ± 3.3	58.4 (56.3–60.5)	12.4 ± 3.2	13.8 ± 3.1	61.0 (55.5–66.5)
qSOFA	1.9 ± 0.8	2.0 ± 0.8	54.8 (52.8–56.8)	2.0 ± 0.8	2.0 ± 0.8	50.3 (44.9–55.7)
APACHE III	95.1 ± 29.5	107.5 ± 28.3	62.1 (60.0–64.2)	98.3 ± 26.2	109.7 ± 27.6	62.1 (56.6–67.7)
MOSAIC	18.8 ± 10.6	22.4 ± 11.0	59.5 (57.4–61.7)	16.9 ± 10.2	19.2 ± 10.6	56.0 (50.2–61.7)
Day 3 ^b^ (*n* = 2119)						
SOFA	13.4 ± 3.5	15.2 ± 3.2	64.7 (62.3–67.1)	11.4 ± 3.2	14.1 ± 3.3	71.3 (65.5–77.1)
qSOFA	1.6 ± 0.8	1.9 ± 0.8	60.2 (57.9–62.5)	1.5 ± 0.8	2.0 ± 0.7	67.2 (61.3–73.0)
APACHE III	82.7 ± 29.7	100.8 ± 29.4	67.0 (64.6–69.3)	83.6 ± 23.6	104.9 ± 30.1	70.1 (64.2–76.0)
MOSAIC	16.7 ± 10.4	20.6 ± 10.3	61.2 (58.8–63.7)	13.0 ± 8.6	17.9 ± 9.7	64.3 (57.8–70.7)
Day 7 ^c^ (*n* = 1677)						
SOFA	10.8 ± 4.8	14.5 ± 3.7	71.8 (69.3–74.3)	10.0 ± 3.9	13.4 ± 3.9	73.2 (66.5–79.8)
qSOFA	1.2 ± 0.9	1.7 ± 0.8	64.9 (62.4–67.5)	1.4 ± 0.8	1.7 ± 0.8	59.4 (52.3–66.5)
APACHE III	63.5 ± 32.0	90.2 ± 28.0	73.5 (71.0–76.0)	70.8 ± 27.0	91.6 ± 25.4	70.7 (63.7–77.7)
MOSAIC	10.5 ± 9.3	16.8 ± 10.0	68.7 (66.1–71.3)	7.9 ± 7.0	14.7 ± 8.4	74.7 (67.9–81.4)

CRRT, continuous renal replacement therapy; AUC, area under the curve; CI, confidence interval; SOFA, Sequential Organ Failure Assessment; qSOFA, quick Sequential Organ Failure Assessment; APACHE, Acute Physiology and Chronic Health Evaluation; MOSAIC, Mortality Scoring system for AKI with CRRT. ^a^: the missing components of the score system were imputed by a score of 0; ^b^: the missing components of the score system were imputed using the score at day 1 at CRRT; otherwise, they were imputed by a score of 0; ^c^: the missing components of the score system were imputed using the score at day 3 at CRRT; otherwise, they were imputed by a score of 0.

## Data Availability

Not applicable.

## Supplementary Materials

The following are available online at https://www.mdpi.com/article/10.3390/jcm10194592/s1, Table S1: Performance of the scoring systems on day 1 of CRRT initiation in predicting 3-day mortality in the entire cohort and in patients older than 80 years, Table S2: The optimal cutoffs and the corresponding sensitivity and specificity in the analysis with an AUC ≥70%.
