# Supplementary material for: Prognostic Performance of Existing Scoring Systems among Critically Ill Patients Requiring Continuous Renal Replacement Therapy: An Observational Study
Source: J Clin Med. 2021 Oct 6;10(19):4592. doi: 10.3390/jcm10194592 (PMC8509572; doi:10.3390/jcm10194592)
Supplement: Supplementary file 1 [file jcm-10-04592-s001.zip › jcm-1398366-supplementary.pdf]

## Supplementary Materials

**Supplemental Table S1**. Performance of the scoring systems on day 1 of CRRT initiation in predicting 3-day mortality in the entire cohort and in patients older than 80 years

| | Total cohort | | | Cohort with octogenarian (Age ≥80 years) | | |
|---|---|---|---|---|---|---|
| Score | Survivor (*n* = 2,119) | Non-survivor (*n* = 1,251) | AUC (95% CI) | Survivor (*n* = 321) | Non-survivor (*n* = 237) | AUC (95% CI) |
| SOFA | 14.3 ± 3.4 | 14.9 ± 3.3 | 54.7 (52.7 - 56.7) | 13.1 ± 3.2 | 13.9 ± 3.2 | 56.3 (51.5 - 61.1) |
| qSOFA | 1.9 ± 0.8 | 2.1 ± 0.8 | 56.1 (54.2 - 57.9) | 2.0 ± 0.8 | 2.0 ± 0.8 | 52.4 (47.9 - 56.8) |
| APACHE III | 99.3 ± 28.9 | 112.1 ± 28.0 | 62.5 (60.5 - 64.4) | 102.4 ± 27.1 | 113.6 ± 27.1 | 62.1 (57.4 - 66.7) |
| MOSAIC | 19.7 ± 10.6 | 24.3 ± 11.2 | 61.5 (59.5 - 63.4) | 16.9 ± 10.3 | 21.2 ± 10.5 | 62.1 (57.4 - 66.7) |

CRRT, continuous renal replacement therapy; AUC, area under the curve; CI, confidence interval; SOFA, Sequential Organ Failure Assessment; qSOFA, quick Sequential Organ Failure Assessment; APACHE, Acute Physiology and Chronic Health Evaluation; MOSAIC, Mortality Scoring system for AKI with CRRT

a: the missing components of the score system were imputed by a score of 0.

**Supplemental Table S2**. The optimal cutoffs and the corresponding sensitivity and specificity in the analysis with an AUC ≥70%.

| Day of mortality | Score | Day after CRRT | Cohort | AUC, % (95% CI) | Cutoff | Sensitivity, % (95% CI) | Specificity, % (95% CI) |
|---|---|---|---|---|---|---|---|
| 7-day | APACHE III | 3rd day | Total | 76.1 (73.6 - 78.5) | 107 | 63.8 (59.1 - 68.3) | 75.0 (72.8 - 77.1) |
| 7-day | qSOFA | 3rd day | ≥80 years | 74.3 (68.8 - 79.7) | 2 | 90.0 (81.9 - 95.3) | 43.3 (36.5 - 50.2) |
| 7-day | APACHE III | 3rd day | ≥80 years | 78.9 (73.1 - 84.8) | 108 | 67.8 (57.1 - 77.3) | 78.6 (72.5 - 83.9) |
| ICU | SOFA | 7th day | Total | 74.1 (71.7 - 76.5) | 14 | 67.3 (63.5 - 71.0) | 67.6 (64.5 - 70.6) |
| ICU | APACHE III | 7th day | Total | 74.7 (72.3 - 77.1) | 82 | 65.5 (61.6 - 69.3) | 71.0 (67.9 - 73.9) |
| ICU | MOSAIC | 7th day | Total | 71.3 (68.8 - 73.9) | 10 | 77.6 (74.1 - 80.9) | 54.0 (50.7 - 57.2) |
| ICU | SOFA | 7th day | ≥80 years | 75.4 (68.9 - 81.8) | 13 | 70.2 (59.3 - 79.7) | 70.4 (61.6 - 78.2) |
| ICU | APACHE III | 7th day | ≥80 years | 72.6 (65.8 - 79.3) | 85 | 65.5 (54.3 - 75.5) | 71.2 (62.4 - 79.0) |

| | | | | | | | |
|---|---|---|---|---|---|---|---|
| ICU | MOSAIC | 7th day | ≥80 years | 74.2 (67.5 - 80.9) | 13 | 60.7 (49.4 - 71.2) | 78.4 (70.2 - 85.3) |
| In-hospital | SOFA | 7th day | Total | 71.8 (69.3 - 74.3) | 12 | 78.9 (75.8 - 81.8) | 51.6 (48.1 - 55.1) |
| In-hospital | APACHE III | 7th day | Total | 73.5 (71.0 - 76.0) | 75 | 69.9 (66.4 - 73.2) | 64.9 (61.5 - 68.2) |
| In-hospital | SOFA | 7th day | ≥80 years | 73.2 (66.5 - 79.8) | 13 | 63.3 (53.5 - 72.3) | 73.0 (63.2 - 81.4) |
| In-hospital | APACHE III | 7th day | ≥80 years | 70.7 (63.7 - 77.7) | 82 | 65.1 (55.4 - 74.0) | 67.0 (56.9 - 76.1) |
| In-hospital | MOSAIC | 7th day | ≥80 years | 74.7 (67.9 - 81.4) | 9 | 77.1 (68.0 - 84.6) | 62.0 (51.8 - 71.5) |

AUC, area under the curve; CRRT, continuous renal replacement therapy; CI, confidence interval; ICU, intensive care unit; SOFA, Sequential Organ Failure Assessment; qSOFA, quick Sequential Organ Failure Assessment; APACHE, Acute Physiology and Chronic Health Evaluation; MOSAIC, Mortality Scoring system for AKI with CRRT.
